# Authority Brings Responsibility: Feedback from Experts Promotes an Overweighting of Health-Related Pseudoscientific Beliefs

**DOI:** 10.3390/ijerph192215154

**Published:** 2022-11-17

**Authors:** Josue Garcia-Arch, Itxaso Barberia, Javier Rodríguez-Ferreiro, Lluís Fuentemilla

**Affiliations:** 1 Cognition and Brain Plasticity Group, Bellvitge Institute for Biomedical Research, 08907 Hospitalet de Llobregat, Spain; 2 Department of Cognition, Development and Educational Psychology, Institut de Neurociències (INUB), University of Barcelona, 08035 Barcelona, Spain; 3 Institut de Neurociències (INUB), Universitat de Barcelona (UB), Passeig de la Vall d’Hebron 171, 08035 Barcelona, Spain; 4 Grup de Recerca en Cognició i Llenguatge (GRECIL), Departament de Cognició, Desenvolupament i Psicologia de l’Educació, Secció Processos Cognitius, Universitat de Barcelona (UB), Passeig de la Vall d’Hebron 171, 08035 Barcelona, Spain

**Keywords:** pseudoscience, public health, healthcare practices, belief updating, misinformation

## Abstract

The popularity and spread of health-related pseudoscientific practices is a worldwide problem. Despite being counteracted by competent agents of our societies, their prevalence and spread continue to grow. Current research has focused on identifying which characteristics make us more likely to hold pseudoscientific beliefs. However, how we hold these beliefs despite all the available information against them is a question that remains unanswered. Here, we aimed to assess if the development of health-related pseudoscientific beliefs could be driven by a positive bias in belief updating. Additionally, we aimed to explore whether this bias could be exacerbated, depending on source credibility. In this study, participants (N = 116) underwent a belief updating task where they offered their agreement with various health-related pseudoscientific statements before and after receiving supporting and discrediting feedback from (a) experts (doctors), (b) peers, or (c) a random number generator. Our results suggest that when receiving feedback from experts (but not from peers or random feedback), the participants preferentially integrated supporting information relative to discrediting information about health-related pseudoscience. We discuss the implications of this biased belief updating pattern on health-related pseudoscientific research and suggest new strategies for intervention focused on increasing awareness, training, and consensus among healthcare practitioners.

## 1. Introduction

Alongside the relentless growth in scientific advances, we are experiencing an increase in the amount of disciplines that mimic the characteristics that render science trustworthy: pseudosciences. Far from being innocuous, their capacity to infiltrate and proliferate in the societal tissue has terrible consequences. One of the best examples of the impact of these practices comes from health-related pseudosciences, which have produced unjustified investments of public money [1], dangerous interference with medical treatments [2], and have been associated with a number of deaths in recent years [3]. Due to the need to understand this phenomenon, a body of correlational studies has been growing. To date, the results of these research works suggest that there is a variety of cognitive correlates for pseudoscience endorsement that are mainly rooted in cognitive biases [4]. These beliefs have been associated with well-known phenomena such as illusions of causality [5,6], jump-to-conclusions [7], probabilistic reasoning biases [8], and self-reported measures of intuitive and analytic cognitive styles [8,9]. Unfortunately, correlational studies fail to account for how pseudoscientific beliefs emerge in the population and therefore remain limited in informing of effective strategies to ameliorate them in society. To counteract their proliferation, we need to further our understanding of how these beliefs develop, which requires us to understand how we deal with the pseudoscientific information we receive from our environment. 

Information on health-related pseudoscience is easily accessible [10]. Through TV and social media, we are all exposed to an endless stream of information, with few restrictions. Indeed, the popularity of low-value healthcare practices has been partly attributed to the increased availability of information on the internet and the influence of our peers [11]. Of even greater concern is the fact that health-related pseudoscience is sometimes promoted by those who should be the standard bearers of health sciences; namely, healthcare professionals. Current evidence suggests that a considerable proportion of physicians have at some point recommended the use of non-evidence-based treatments [12,13]. Studies also suggest that some practitioners have recommended such treatments without even having received formal instruction in relation to them [13,14], and that such recommendations were often based on their personal beliefs [14]. This lack of rigorousness might become a serious problem, particularly considering the asymmetry between practitioners and patients. Healthcare professionals are the custodians of some of the most valuable knowledge for human beings, such as the potential causes and remedies for adverse medical conditions. As such, they represent a strong source of authority, power, and trust, which significantly influence patients’ decision making [15,16]. Therefore, as well as improving the acceptance of evidence-based interventions [16], healthcare professionals may also promote pseudoscience endorsement, which may hinder the efforts to reduce the proliferation of these practices.

In the face of the growth and danger of health-related pseudoscience, numerous initiatives have emerged to combat them. Health professionals have discredited many of these practices and criticized their promoters [1]; governments and scientific societies have launched campaigns [12,17]; and private companies have helped to curb the spread of misinformation [18]. Unfortunately, pseudoscientific practices are still highly prevalent nowadays, and how health-related pseudoscientific beliefs are growing despite being actively counteracted by competent and trustworthy agents of our society is a question that remains unanswered.

Evidence from experimental psychology suggests that we can hold a belief and seek information that confirms it as long as it has a positive impact on our feelings or we believe it is useful for us, i.e., if it brings us some benefit, real or perceived [19,20]. However, what benefit is there for us in believing in the effectiveness of pseudoscientific treatments? An intuitive reason might be that they give us hope and increase our sense of control [21,22], just as evidence-based treatments do. We want to believe that if we are trapped in some medical condition, there will be a treatment that will release us from it [23]. Therefore, any information that reinforces the effectiveness of a proposed cure is potentially desirable, even if it comes from pseudoscience [24,25]. Indeed, it has been argued that optimism about the effectiveness of an intervention can lead governments to promote pseudoscientific or poorly supported empirical practices, even when there is evidence against them [1].

From a rational point of view, disregarding opposing evidence for a pseudoscientific treatment just because we want to believe in its effectiveness would be suboptimal. However, when forming and updating our beliefs, we tend to under-weigh undesirable information relative to desirable information (i.e., we prefer “good news”; [26]). It has been suggested that this tendency is motivated by the need to modulate psychological factors such as perceived control, perceived vulnerability, stress, or anxiety [27,28,29]. Interestingly, all these factors have been suggested to be part of people’s psychological reactions to several medical conditions, against which pseudoscientific treatments are supposed to provide us with protection [30]. This places beliefs in health-related pseudoscience to fall within the range of beliefs that are more likely to show a positively biased belief updating. Although this possibility has never been studied experimentally, there is evidence that supports this notion. For example, some patients tend to accept pseudoscientific treatments and avoid information that may discredit them [31]. Moreover, a recent study revealed that having received supporting, health-related, pseudoscientific information was a positive predictor of the participants’ pseudoscientific beliefs, whereas receiving discrediting information showed null predictive capacity [9]. This evidence highlights the importance of studying how health-related pseudoscientific beliefs are updated after receiving supporting and discrediting information.

In this study, we aimed to assess if the development of health-related pseudoscientific beliefs could be driven by a positive bias in belief updating. Additionally, we aimed to explore whether this bias could be exacerbated, depending on source credibility (Experts vs. Peers). In our study, participants were asked to make subjective judgments about the effectiveness of various health-related pseudoscientific proposals before and after receiving pseudo-random supporting (desirable) and discrediting (undesirable) feedback. The participants were divided into three groups, and each group was respectively informed that feedback would be received from (1) experts (doctors), (2) peers (other participants), or (3) a random number generator. We hypothesized that the participants would adjust their opinions more in the direction of the supporting (relative to discrediting) feedback coming from an allegedly meaningful source (i.e., from experts and other peers), but not when received from a non-meaningful one (i.e., a random number generator). 

## 2. Materials and Methods

### 2.1. Participants

The required sample size for the present study was determined by a power analysis conducted in G*Power [32]. Prior related research has found large effect sizes for the positive bias (η^2^ > 0.1) [33], which require a sample size of n = 20 in order to obtain an acceptable power of 80% in repeated measures testing between conditions. However, the assessment of our hypothesis relied on an interaction effect (the positive bias should emerge only in the Experts and Peers groups). We therefore decided to conduct a power analysis for the interaction of between (groups)–within (conditions) factors. We assumed a rather conservative small-to-moderate interaction effect of η^2^ = 0.03. The power analysis estimated that for an acceptable power of 80%, we would need at least 81 participants. The number of participants that participated in our study was 137 (undergraduate students, 70.07% women, M_age_ = 20.85, SD_age_ = 3.74, 95% CI [20.21, 21.49]). The participants were recruited through a convenience sampling. They were invited to participate in the experiment via an online platform for experimental research pertaining to the University of Barcelona (http://www.ub.edu/psicologiabasica/; accessed on 14 July 2022).

The participants were randomly divided into 3 groups based on 3 different experimental conditions. At the start of the experiment, the program randomly assigned each participant (full randomization) to one of the experimental groups. The participants in the Experts group (n = 45) were told that they were receiving feedback from experts (doctors). Those in the Peers group (n = 47) were presented with feedback allegedly coming from other participants in the experiment, and those in the Random group (n = 45) were told that they were receiving random feedback. Prior to analyzing the data, we applied two exclusion criteria. Following earlier literature [33], the first exclusion criterion was based on scores on the Beck Depression Inventory (BDI-II) [34]. Thirteen participants (9.48% of the total sample) with moderate or severe depressive symptoms (BDI-IIscore > 18) were excluded (6 in Experts, 5 in Peers, 2 in Random). Participants (n = 8; 3 in Experts, 1 in Peers, 4 in Random) who did not complete at least 80% of the trials of the experimental task were also excluded from further analysis. Thus, the final sample included 116 participants (nExperts = 36, nPeers = 41, nRandom = 39). All participants provided written informed consent. The participants received course credit for their participation.

### 2.2. Procedure

#### 2.2.1. Stimuli

Stimuli consisted of a set of 30 pseudoscientific statements related to health improvements, health interventions, and prevention of health problems (e.g., “Chiropractic (treatment of the musculoskeletal system) can improve our immune system”, “Natural remedies, such as Bach flower remedies, help to overcome emotional imbalances”, “Massage on the circulatory system (lymphatic drainage) can help to improve the body’s defenses”, see Table S1, Supplementary Files). These stimuli were extracted from psychometrically validated questionnaires [6,9,35] and complemented with new items based on an official list of pseudoscientific interventions recently published by the Spanish government (https://www.conprueba.es/; accessed on 10 September 2021).).

#### 2.2.2. Experimental Task

**Figure 1:**
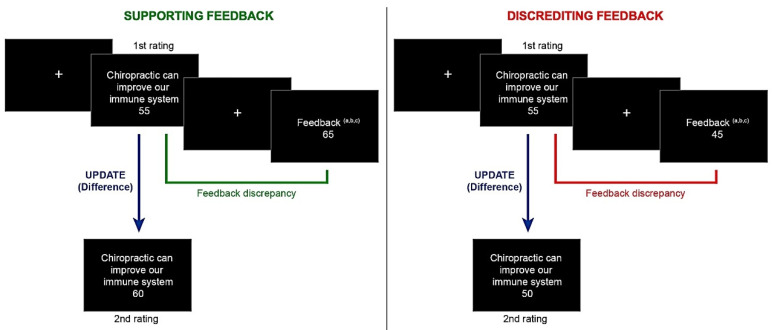
Belief updating task. (**Left**) Example of a supporting (desirable) feedback trial; participants’ initial rating is lower than the feedback received. In each trial, participants had to provide an initial judgement responding to a general question (“How much do you believe the following statement is true from 1 to 100?”). Feedback indices (a,b,c) represent differences in the statements presented depending on the feedback group. (a, Experts) “On average, experts believe that this statement is true from 1 to 100 per cent: ”; (b, Peers) “On average, other participants of the experiment believe that this statement is true from 1 to 100 per cent: ”; (c, Random) “The random number for this trial is: ”. In green, feedback discrepancy represents the difference between the participants’ initial rating and the feedback received. In blue, update (dependent variable) is represented as the difference between the first and second assessments (see Experimental Task section for details). (**Right**) Example of a discrediting (undesirable) feedback trial; participants’ initial rating is higher than the feedback received. In red, feedback discrepancy represents the (discrediting) difference between the participants’ initial rating and the feedback received.

The experimental procedure followed the general lines of a well-known experimental belief updating task, which has been widely used and adapted to study the processing of different feedback types [26,36]. In each trial, the participants read a pseudoscientific claim displayed on the screen. They then had 5 s to provide a rating (first rating) for their level of agreement with the provided statement, using a scale ranging from 1 (indicating absolute disagreement) to 100 (indicating absolute agreement). Pseudoscientific claims were presented in random order. After a fixation cross (1 s), the participants were shown, for 4 s, what they believed to be (a) the average ratings of 8 experts on the same claim (Experts group), (b) the average ratings given by other participants in the experiment on the same claim (Peers group), or (c) a random number (Random group) (see Note S1, Supplementary Files, for the original and translated instructions). This process was repeated for all 30 pseudoscientific statements, with a fixation cross (1 s) after every trial. After completing this process for every pseudoscientific claim, the participants again provided their belief ratings for each statement (second rating). The experimental procedure is outlined in Figure 1.

The target measure of this task is the difference between the first and the second rating (i.e., update). Updating scores were computed as follows: If participants received supporting feedback (the feedback received was higher than participants’ rating), the update was computed as the difference between the second and the first rating (rating 2 (post feedback)–rating 1 (pre feedback)). If participants received discrediting feedback (the feedback received was lower than participants’ rating), the update was computed as the difference between the first and the second rating (rating 1 (pre feedback)–rating 2 (post feedback)). As in previous studies, we also computed a variable aimed to capture the difference between one’s own first rating and the feedback received, namely feedback discrepancy [36].

To control for potential confounds, we introduced complementary measures as covariates, such as familiarity (how familiar each statement was, on a scale from 1 to 4), memory accuracy (computed as the average difference between the participants’ memory of the feedback received for each statement and the actual feedback, in absolute value), and prior experience (participants had to answer whether they themselves or through a professional or acquaintance had put into practice any of the pseudoscientific proposals presented to them; they were asked to provide a dichotomous response: Yes or No). The selection of covariates was based on prior studies [29,36]. At the end of the experiment, after collecting the participants’ data on the covariates and BDI-II, they were informed that the feedback received was in fact pseudo-random, and that all claims presented were considered to be pseudo-scientific proposals.

#### 2.2.3. Feedback Generation

As in previous studies [36], feedback was pseudo-randomly generated by a random number generator. To create the feedback conditions (supporting and discrediting), the random number generator was restricted to evenly generate higher (supporting; 50%) and lower (discrediting; 50%) scores than the participants’ ratings. For each rating in the supporting condition, the program randomly chose a score between the participants’ rating (+1) and the upper limit of the scale (100). In the discrediting condition, the program randomly chose a score between the participants’ rating (−1) and the lower limit of the scale (1).

We restricted the maximum absolute difference between the feedback and the participants’ ratings to 40 (out of 99).

## 3. Results

In this research, we hypothesized that participants from the Experts and Peers groups would exhibit a positively biased belief updating in the task, that is, the participants’ beliefs would adjust more to supporting feedback than to discrediting feedback. To test this hypothesis, we conducted a 3 (Group: “Experts”, “Peers”, and “Random”) by 2 (Feedback Condition: “Supporting”, “Discrediting”) Repeated Measures Analysis of Variance (RM ANOVA). Update (belief pre/post-change) was set as the dependent variable, and Group, Feedback Condition, and the interaction between the two were entered as between and within subjects’ factors, respectively. Post hoc contrasts were Bonferroni-corrected.

**Figure 2:**
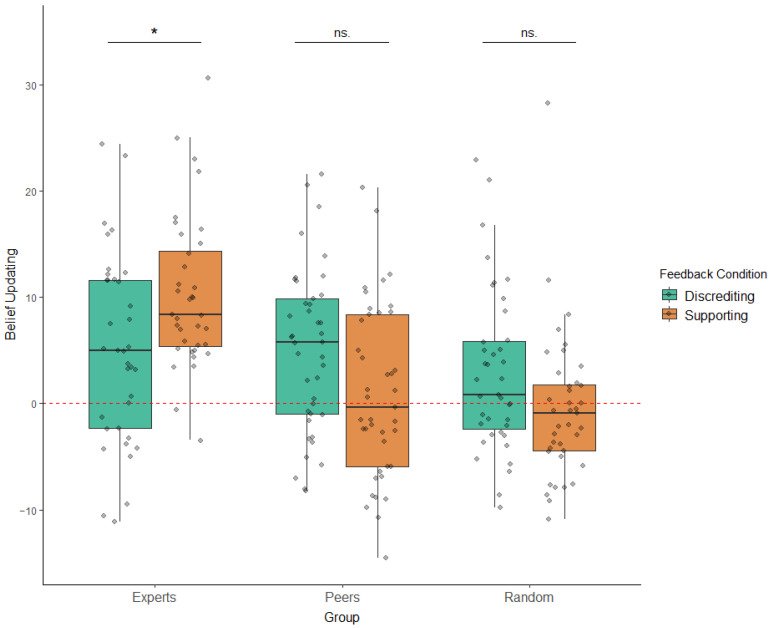
Differences in average belief updating between groups (*x*-axis) and conditions (colors). Dashed red line denotes “no update” (0). Jittered points represent individual averages. Color-blind friendly palette. Statistical significance codes: * *p* < 0.05 (Bonferroni-corrected).

Results from the RM ANOVA, including all covariates (feedback discrepancy, familiarity, participants’ initial beliefs, prior experience, and memory accuracy) revealed a significant main effect for the Group factor: F(2,220) = 13.14, *p* < 0.001, ηp2 = 0.11; a non-significant main effect for the Feedback Condition: F(2,220) = 0.92, *p* = 0.3391, ηp2 = 0.004; and a significant interaction between Group and Feedback Condition: F(2,220) = 8.26, *p* < 0.001, ηp2 = 0.07 (Figure 2). The results obtained without the inclusion of the mentioned covariates yielded similar results (Group: F(2,225) = 13.10, *p* < 0.001, ηp2 = 0.1; Feedback: F(2,225) = 0.921, *p* = 0.3392, ηp2 = 0.004; Group-by-Feedback Condition interaction: F(2,225) = 8.24, *p* < 0.001, ηp2 = 0.07).

To scrutinize the results obtained, we then tested whether the interaction effect remained significant when restricting the analysis to Experts vs. Peers (contrast 1), Experts vs. Random (contrast 2), and Peers vs. Random (contrast 3). All contrasts were Bonferroni-corrected. The results of contrast 1 showed a significant main effect for Group: F(1,144) = 11.83, *p* = 0.0082, ηp2 = 0.08; a non-significant main effect for Feedback Condition: F(1,144) = 0.041, *p* = 1, η2 = 0.0002; and a significant interaction between Group and Feedback Condition: F(2,144) = 11.29, *p* = 0.00891, ηp2 = 0.07. The results of contrast 2 showed a significant main effect for Group: F(1,140) = 25.73, *p* < 0.001, ηp2 = 0.16; a non-significant main effect for Feedback Condition: F(1,140) = 0.32, *p* = 1, ηp2 = 0.002; and a significant interaction between Group and Feedback Condition: F(2,140) = 12.37, *p* = 0.0045, ηp2 = 0.08. The results of contrast 3 showed a non-significant main effect for Group: F(1,151) = 2.42, *p* = 0.360, ηp2 = 0.02; a significant main effect for Feedback Condition: F(1,150) = 9.78, *p* = 0.018, ηp2 = 0.06; and a non-significant interaction between the two: F(1,151) = 0.03, *p* = 1, ηp2 = 0.002. Subsequent post hoc analyses revealed that the participants updated their beliefs more significantly after receiving supporting feedback as compared to discrediting feedback in the Experts group, F(1,64) = 7.41, *p* = 0.02433, ηp2 = 0.1 (Supporting feedback: M = 10.235, SD = 7.183, 95% CI[7.804, 12.665]; Discrediting feedback: M = 5.041, SD = 8.946, 95% CI[2.013, 8.067]). However, no significant differences in updates between the feedback conditions were found either in the Peers group, F(1,74) = 5.14 *p* = 0.0792, ηp2 = 0.06 (Supporting feedback: M = 1.002, SD = 8.121, 95% CI[−1.561, 3.565]; Discrediting feedback: M = 5.068, SD = 7.658, 95% CI[2.651, 7.481]) or in the Random group, F(1,70) = 4.61, *p* = 0.105, η2 = 0.06 (Supporting feedback: M = 10.235, SD = 7.183, 95% CI[7.804, 12.665]; Discrediting feedback: M = −0.578, SD = 6.959, 95% CI[−2.834, 1.678]) (Figure 2). Overall, these results reveal that Supporting feedback produced greater updates than Discrediting feedback only in the Experts group.

### Control Analysis

In what follows, we provide additional statistical assessments to confirm that differences between groups were not partially driven by the differences in nuisance variables, such as familiarity, memory accuracy, and prior experience, in relation to the pseudoscientific statements. Firstly, we tested whether feedback discrepancies were not systematically biased between feedback conditions and across groups. The results showed that feedback discrepancies did not significantly differ, neither across groups: F(2,225) = 0.31, *p* = 0.732, ηp2 = 0.002, nor across feedback conditions: F(2,225) = 0.23, *p* = 0.628, ηp2 = 0.001, and that the Group × Feedback interaction was not significant: F(2,225) = 1.084, *p* = 0.3401, η2 = 0.01. The same analysis was conducted for familiarity ratings (Group, F(2,225) = 2.90, *p* = 0.0577, ηp2 = 0.03; Feedback Condition, F(2,225) = 0.033, *p* = 0.855, ηp2 = 0.0001; Group-by-Feedback Condition interaction, F(2,225) = 0.36, *p* = 0.699, η2 = 0.003), memory accuracy (Group, F(2,225) = 0.502, *p* = 0.605, ηp2 = 0.004.; Feedback Condition, F(2,225) = 1.69, *p* = 0.194, ηp2 = 0.007; Group-by-Feedback Condition interaction, F(2,225) = 2.46, *p* = 0.087, ηp2 = 0.02), and prior experience (Group, F(2,225) = 1.34, *p* = 0.263, ηp2 = 0.01.; Feedback Condition, F(2,225) = 0.0002, *p* = 0.999, ηp2 < 0.001; Group-by-Feedback Condition interaction, F(2,225) < 0.001, *p* = 0.999, η2 < 0.001), with similar results.

## 4. Discussion

The aim of the present study was to assess if the development of health-related pseudoscientific beliefs could be explained by a positive bias in belief updating. To this end, we conducted an experiment in which the participants received both supporting (desirable) and discrediting (undesirable) information regarding various health-related pseudoscientific proposals. We included three different sources of information: experts (doctors), peers (other participants), and a random number generator. We found that only the participants who received feedback from experts incorporated more supporting (relative to discrediting) information into their health-related pseudoscientific beliefs. These findings indicate that the experts’ feedback can trigger a positive bias in belief updating, which may critically contribute to the acceptance of health-related pseudoscience.

To the best of our knowledge, this is the first study to experimentally investigate whether the updating of pseudoscientific health beliefs is positively biased after receiving supporting and discrediting information from different sources of information. Our results go beyond the identification of which individuals are most likely to show greater adherence to pseudoscience [6,7,8,9] and suggest that supporting and discrediting information provided by expert practitioners is differently weighted by the non-professional population. More specifically, our work indicates that when receiving feedback from experts about pseudoscientific treatments, people underweight discrediting information relative to supporting information. That is, when receiving experts’ feedback about the effectiveness of dubious healthcare-related proposals, we prefer “good news”. These findings are consistent with the proposal that valenced beliefs are updated in a valence-dependent manner [26,37], which has been linked to the need to preserve personal well-being [27,28,29]. This phenomenon may help us to understand why the resistance, spread, and reach of pseudoscience—despite efforts by scientists and institutions to discredit harmful and non-scientific practices—still manage to have an influence on policies and state funding [1].

Intriguingly, we found a positive bias in belief updating in the group of participants that received feedback from experts, but not in the one that received feedback from peers. In the context of this study, a plausible interpretation of this finding is that participants may have considered the feedback provided by peers not relevant enough to trigger meaningful belief updating. Our results provide some evidence in favor of this suggestion, given that contrasts between Peers and Random groups yielded similar results. This is not completely surprising, given that peers, unlike expert practitioners, are unlikely to have deep and meaningful knowledge of the targeted beliefs in this study [15,16]. This is contrary to what happens with beliefs related to one’s own personal identity, where feedback from peers is fundamental to self-concept updating [36]. Indeed, recent work has suggested that when seeking information, we first estimate what this information will reveal to us and then compute its expected value in terms of its impact on our cognition, affect, and actions [38]. The lack of authority and knowledge of peers in this context may reduce the expected impact of this information, thus making it irrelevant. However, we cannot rule out the possibility of obtaining a different result if other sources of feedback from the participants’ social lives were included, such as known peers, friends, or family members who might not only be more relevant but also more authoritative, depending on their role in the participants’ lives. Future research should address this possibility to better understand the impact of this source of information on the recipients’ updating of pseudoscientific health beliefs.

Unexpectedly, we found a main effect of feedback valence when comparing the results of the groups who were told that feedback proceeded from other peers with those who were told that feedback was randomly generated by an algorithm. This finding may indicate that the participants in these two groups tended to update their beliefs to a greater extent when receiving feedback that discredited rather than supported their initial judgment. However, these results should be taken with caution because a subsequent post hoc comparison of the degree of update for each group as a function of type of feedback did not reach statistical significance. This opens the possibility of studying whether even non-meaningful, discrediting feedback could make participants re-evaluate their beliefs when they are re-exposed to pseudoscientific claims, thus resulting in the tendency to reduce their own beliefs.

The results obtained from the Experts group suggest that positively biased belief updating could be an underlying cognitive mechanism for the acceptance of health-related pseudoscience, which emphasizes the relevance of the source of information. More generally, they also reveal the potential dangers of providing non-scientific information in a situation where there are strongly unbalanced roles in terms of power and knowledge, even when discrediting evidence is available. The dangers of the positively biased belief updating triggered in response to expert feedback may also be exacerbated by different cultural and socio-demographic characteristics. Although the power and authority of doctors are recognized worldwide [39], people with lower incomes and lower levels of education are more likely to assign greater authority to them [40]. In turn, lower levels of education have already been linked to greater acceptance of pseudoscientific proposals [9]. Overall, economically disadvantaged populations with less access to education may be particularly vulnerable to the ethical violations posed by the spread of pseudoscientific proposals in the healthcare domain. The population of patients with critical and/or chronic health conditions may also require special attention. There is evidence that patients with severe medical conditions seek non-scientific remedies and even hide it from their physicians [30]. Their strong need for a cure and the lack of effectiveness of conventional medicine may accentuate their tendency to preferentially incorporate information that is supportive of a new treatment, even if it lacks scientific support. Physicians dealing with this population of patients should be vigilant and help them to evaluate the sources of information that they consult as well as make them aware of the functioning of our cognition when receiving information that, although unreliable, is convenient for us.

More importantly, although the mass transmission of misinformation is a fact [10], our findings suggest that the information we receive from supposedly reliable agents may be the key trigger or main accelerator of beliefs in health-related pseudoscience. However, the work of policy makers against the propagation of misinformation is not only hampered by a lack of training or consensus among health professionals; today, the double-edged global interconnectedness offered by new technologies promotes the rapid transmission of misleading information, which is very difficult to monitor [41]. Moreover, virtual environments make it difficult to assess the credibility of information sources, which can influence thousands of opinions and may trigger belief updating biases despite not possessing great power or authority in the health domain [42]. New measures involving the collaboration of social media companies as well as competent figures in the health field should be developed in the coming years to help curb the pervasive effects of misinformation.

A recent work has suggested that in the general population, there is an overestimation of the proportion of physicians who do not adhere to scientifically supported practices [16]. This study also suggested that such beliefs can be intervened upon to encourage evidence-based treatment choices. Our results complement these findings by suggesting that the neutralization of practitioners’ propagation of non-scientific advice may be crucial to diminishing the population’s beliefs about doctors’ pseudoscience acceptance, and pseudoscience acceptance in itself. However, implementing strategies to increase consensus and scientific rigor among health professionals might be particularly complex in specific situations. A clear example of this is the recent COVID-19 pandemic. Despite great and rapid scientific advances, the population’s pressing need for constant advice and guidance generated a situation where concern grew faster than rigorous information. In turn, the lack of standardized scientific information and professional preparation [43], the initial lack of uniformity of medical criteria [44], and the fact that healthcare professionals were also affected by the pandemic as non-professional individuals [45] created the ideal substrate for the spread of misleading information among practitioners and the general population [46,47]. To prevent a spike in the transmission of misleading health-related information in future crisis situations, preventive protocols and guidelines should be developed for times when scientific knowledge is temporarily limited.

## 5. Conclusions

The current situation regarding the spread and acceptance of health-related pseudoscience is worrying and out of control [1,10,12]. Our study suggests that our positive biases are awaiting the arrival of “reliable” sources of information to contribute to their acceptance. Our findings suggest that countermeasures aimed at the development of critical thinking in the general population [48] need to be complemented with new strategies to ensure the comprehensive and updated training of health professionals. More importantly, to prevent global health-related misinformation, the vulnerability of different populations to the acceptance of misleading medical information, the influence of transmission channels, and the potential difficulty of managing crisis situations with limited scientific knowledge should be taken into account. Curbing the spread of pseudoscience requires a shared endeavor. As non-professionals, we should bear in mind that being inexperienced in a field does not exempt us from having the capacity to evaluate the sources of information that help us make decisions, nor does it preclude us from being aware of our own biases. As professionals, we should be aware of the consequences of our work, especially when derailing from deontological principles. Just as our ignorance does not exempt us from duty, our authority brings responsibility.

## Data Availability

The dataset analyzed during the current study is available at the Open Science Framework [https://osf.io/mnywk/?view_only=25f34bb9ef2e45838eecb922d65ec480; accessed on 13 July 2022].

## Supplementary Materials

The following supporting information can be downloaded at: https://www.mdpi.com/article/10.3390/ijerph192215154/s1, Table S1: List of pseudoscientific statements and descriptive statistics of participants’ initial belief ratings, Note S1: Feedback Instructions.
