# Supplementary material for: Authority Brings Responsibility: Feedback from Experts Promotes an Overweighting of Health-Related Pseudoscientific Beliefs
Source: Int J Environ Res Public Health. 2022 Nov 17;19(22):15154. doi: 10.3390/ijerph192215154 (PMC9690443; doi:10.3390/ijerph192215154)
Supplement: Supplementary file 1 [file ijerph-19-15154-s001.zip › ijerph-2020173-supplementary.pdf]

Supplementary Materials

Table S1. List of pseudoscientific statements and descriptive statistics of participants' initial belief ratings (means and standard deviations).

Item	M	SD
Through reflexology (manipulation of energy channels through the soles of the feet) medical problems such as migraine can be treated.	36.97	32.09
Some autoimmune diseases can be treated with substances from bees (honey, propolis, etc.).	42.81	28.99
By controlling the energy of the mind we can influence physical conditions (psychic healing).	40.51	33.02
By manipulating specific pressure points on the body, physical and psychological ailments can be treated.	54.30	31.70
Various diseases can be treated by analysis of iris colour and administration of certain amino acids.	29.13	29.93
By applying certain minerals to the skin, inflammation of a particular area can be reduced.	39.58	28.89
Changing the way we write (graphotherapy) can help to treat emotional problems.	23.35	26.64
Orange juice is an effective remedy for fighting cold viruses.	42.23	31.42
A positive and optimistic attitude towards life helps to prevent cancer.	34.49	32.85
Intestinal lavage with water and other compounds (hydrocolonotherapy) helps to treat problems such as irritable bowel syndrome.	46.01	28.58
Hot stone massage can help eliminate toxins and boost the body's metabolism.	36.10	29.37
Massage on the circulatory system (lymphatic drainage) can help to improve the body's defences.	50.70	30.72
The use of vitamin-based supplements helps prevent fractures as we grow older.	48.34	29.04
Treatment with plants with psychotropic properties helps to cure medical ailments.	53.95	29.65
Through the use of energy (reiki), people have been cured of physical illnesses.	30.29	29.21
In some cases, being exposed to magnetic fields is positive for health.	23.59	26.00
Finding the emotional source of a problem can influence the state of physical health.	72.97	20.38
There are therapies based on the use of the sea (thalassotherapy) capable of preventing and improving different medical conditions.	40.91	28.30
Oxygen-based therapies are capable of treating degenerative diseases.	40.85	30.89
There are therapies based on the rebalancing of internal energy that can improve the state of health.	40.68	33.24

The removal of metals from the body by means of substances that bind to them improves atherosclerosis.	33.54	25.66
Improving relationships with our environment can have an impact on the recovery from lung conditions.	43.12	30.40
Osteopathy is able to induce the body to heal itself by manipulating muscles and bones.	42.50	33.06
Chiropractic (treatment of the musculoskeletal system) can improve our immune system.	44.78	28.08
With natural teas and/or infusions, the same symptoms can be treated as with conventional medicines.	23.05	27.17
Detox diets or therapies are effective in eliminating toxic substances from the body.	45.16	31.94
Eating an alkaline diet (based on pH control) can prevent serious diseases.	49.98	30.34
Homeopathic remedies are effective as complements in the treatment of some diseases.	36.73	28.98
The superficial insertion of needles into specific parts of the body can be used to treat pain problems.	54.77	30.29
Natural remedies, such as Bach flower remedies, help overcome emotional imbalances.	31.64	28.69

Note S1:

Feedback instructions:

Experts

(Original): Después de cada valoración que proporcione sobre su grado de acuerdo con cada una de las afirmaciones, se le presentará una nueva información. Esta información es la media de las valoraciones que 6 expertos han realizado sobre su grado de acuerdo con las mismas afirmaciones que se le presentarán. Los expertos que han participado en este estudio han sido: 3 médicos de familia, 1 traumatólogo y 2 médicos residentes.

(Translated): After each rating you provide regarding your agreement with each of the statements, you will be presented with new information. This information is the average of the evaluations that 6 experts have made regarding their degree of agreement with the same statements that will be presented to you. The experts involved in this study were: 3 family doctors, 3 traumatologist and 2 resident doctors.

Peers

(Original): Después de cada valoración que proporcione sobre su grado de acuerdo con cada una de las afirmaciones, se le presentará una nueva información. Esta información es la media de las valoraciones que 8 participantes que ya han participado y completado este estudio han hecho sobre las mismas afirmaciones que se le presentarán a usted.

(Translated): After each rating you provide regarding your agreement with each of the statements, you will be presented with new information. This information is

the average of the ratings that 8 participants who have already participated and completed this study have made on the same statements that will be presented to you.

Random

(Original): Después de cada valoración que proporcione sobre su grado de acuerdo con cada una de las afirmaciones, se le presentará una nueva información. Esta información es sólo un número que el programa elegirá al azar.

(Translated): After each rating you provide regarding your agreement with each of the statements, you will be presented with new information. This information is just a number that the program will randomly choose.
